# Acceptability of pulmonary rehabilitation in Malawi: a qualitative study

**DOI:** 10.1136/bmjresp-2024-002547

**Published:** 2025-05-22

**Authors:** Fanuel Meckson Bickton, Talumba Mankhokwe, Beatrice Chavula, Emily Chitedze, Martha Manda, Cashon Fombe, Martha Mitengo, Langsfield Mwahimba, Moses Isiagi, Richard N. van Zyl-Smit, Susan Hanekom, Martin Heine, Harriet Shannon, Jamie Rylance, Enock Chisati, Stephen B. Gordon, Felix Limbani

**Affiliations:** 1 Malawi-Liverpool-Wellcome Trust Clinical Research Programme, Blantyre, Malawi; 2 Department of Rehabilitation Sciences, School of Life Sciences and Allied Health Professions, Kamuzu University of Health Sciences, Blantyre, Malawi; 3 Physiotherapy Department, Queen Elizabeth Central Hospital, Blantyre, Malawi; 4 Division of Global Surgery, Department of Surgery, Groote Schuur Hospital, University of Cape Town, Cape Town, South Africa; 5 Division of Pulmonology and Department of Medicine, University of Cape Town & Groote Schuur Hospital, Cape Town, South Africa; 6 Division of Physiotherapy, Department of Health and Rehabilitation Sciences, Faculty of Medicine and Health Sciences, Stellenbosch University, Cape Town, South Africa; 7 Julius Global Health, Julius Center for Health Sciences and Primary Care, University Medical Centre Utrecht, Utrecht University, Utrecht, The Netherlands; 8 Institute of Sport and Exercise Medicine, Faculty of Medicine and Health Sciences, Stellenbosch University, Cape Town, South Africa; 9 UCL Great Ormond Street Institute of Child Health, University College London, London, UK

**Keywords:** Pulmonary Rehabilitation

## Abstract

**ABSTRACT:**

**Background:**

Pulmonary rehabilitation (PR) is an effective non-pharmacological intervention for people with chronic respiratory diseases (CRDs), but its acceptability in Malawi was unknown.

**Objectives:**

To explore patients’ acceptability of PR at Queen Elizabeth Central Hospital, Blantyre, Malawi.

**Methods:**

This was a pre–post cohort study where participants were offered a two times per week hospital-based PR programme for 6 weeks, consisting of endurance and strengthening exercises. Following programme completion, face-to-face semistructured in-depth interviews with the participants were conducted. Interview transcripts were thematically analysed using a deductive approach.

**Results:**

10 adults (five females and five males) out of 14 invited (~70% uptake) participated in the PR programme and subsequent in-depth interviews. Five key themes emerged: (1) debilitating symptom experience of CRD prior to PR; (2) positive impact of PR on living with CRD; (3) contextual programme design improved participants’ experience with PR; (4) one size does not fit all and (5) challenges and opportunities for home-based PR. Participants reported experiencing improvements in physical, psychological and social health associated with PR programme participation. The provision of transport was considered a key facilitator for PR programme completion. Realising the gained PR benefits, participants were willing to continue exercising at their homes.

**Conclusion:**

The PR programme improved the participants’ perceived health status and was well-accepted. Addressing barriers related to transport facilitated immediate implementation while providing a challenge for the scaling and sustainability of PR beyond the project duration. These findings support the drive for shifting chronic care, including rehabilitation, towards primary care and community.

**Trial registration number:**

Prospective; 27 August 2021; ISRCTN13836793.

**WHAT IS ALREADY KNOWN ON THIS TOPIC**

- Pulmonary rehabilitation (PR) is an effective non-pharmacological intervention for people with chronic respiratory diseases (CRDs), but there is a dearth of evidence on its acceptability in Africa.

**WHAT THIS STUDY ADDS**

- This study contributes evidence to PR literature from Africa by investigating its acceptability among people with CRDs in Malawi, a low-income country in southeastern Africa.

**HOW THIS STUDY MIGHT AFFECT RESEARCH, PRACTICE OR POLICY**

- The PR programme in Malawi improved patients’ reported health status and was well-accepted.
- Addressing barriers related to transport would facilitate the scaling up and sustainability of PR beyond the project duration.
- These findings support the drive for shifting chronic care, including rehabilitation, towards primary care and community (thus, community-based or home-based PR).

## Introduction

 Low-income and middle-income countries (LMICs) bear a high burden of the global morbidity and mortality caused by chronic respiratory diseases (CRDs), including asthma, chronic obstructive pulmonary disease (COPD), bronchiectasis and post-tuberculosis lung disease.^1^ Risk factors for CRDs include tobacco smoking, outdoor air pollution, household air pollution, occupational dust exposure and pulmonary tuberculosis (TB).^2^ CRDs are associated with significant chronic morbidity and loss of economic productivity, consequently burdening patients, families and health systems.^3^

A meta-analysis of available literature relating to CRDs in Malawi identified a high burden of chronic respiratory symptoms and abnormal spirometry data (particularly low forced vital capacity) in paediatric and adult populations (including those diagnosed with CRDs, those diagnosed with other diseases such as malnutrition and HIV and those not diagnosed with any disease) from diverse settings (from the community setting to subgroups within an acute clinic).^4^ Notably, even patients who are microbiologically ‘cured’ from TB treatment still experience sequelae such as cough and shortness of breath, impairing their quality of life and placing a continued and sustained financial burden on them and their families.^5 6^

In 2017, CRDs were the third leading cause of death worldwide, behind cardiovascular diseases and neoplasms, with COPD and asthma being the most and second most prevalent CRDs, respectively.^7^ The Global Initiative for Asthma and Global Initiative for Chronic Obstructive Lung Disease guidelines recommend the use of inhaled short-acting beta agonists, inhaled short-acting antimuscarinic agents, inhaled long-acting antimuscarinic agents (LAMA), inhaled long-acting beta agonists (LABA) and LAMA combinations, inhaled corticosteroid (ICS)–LABA combinations, ICS monotherapies, oral methylxanthines, oral leukotriene receptor antagonists, pneumococcal vaccination and phosphodiesterase-4 inhibitors as the mainstay pharmacological therapies in the management of asthma and COPD.^8 9^

However, the poor availability and affordability of these drugs in LMICs,^10^ including in Africa,^11^ limit their impact. Moreover, the development of non-respiratory impairment and functional limitations, which reflect the systemic nature of COPD, are critical determinants of disablement.^12^ Consequently, pharmacotherapy may need to be complemented by comprehensive rehabilitative strategies aimed at the diverse extrapulmonary manifestations of COPD to prevent disability and restore function.^12 13^ Rehabilitation is a set of interventions to address the limitations in everyday physical, mental and social functioning due to ageing or a specific health condition such as chronic disease or injury.^14^ In ‘Rehabilitation 2030: a Call for Action’,^15^ WHO’s Department of Noncommunicable Diseases builds an evidence-based Package of Rehabilitation Interventions, in combination with suggestions for supplying their delivery. Module 4 addresses “cardiopulmonary [cardiorespiratory] conditions”.^16^

For individuals with CRDs, pulmonary rehabilitation (PR) is an essential component of the integrated care.^17^ This is “a comprehensive intervention based on a thorough patient assessment followed by patient-tailored therapies that include, but are not limited to, exercise training, education and behaviour change, which are designed to improve the physical and psychological condition of people with chronic respiratory disease and promote the long-term adherence to health-enhancing behaviours”.^18^ Notably, in people with COPD, PR is supported by high-quality evidence of improvement in symptoms (dyspnoea, fatigue, anxiety, depression), exercise tolerance and overall health-related quality of life.^19^ There is also evidence supporting PR for other CRDs, including interstitial lung disease,^20^ pulmonary hypertension,^21^ asthma,^22^ cystic fibrosis,^23^ bronchiectasis,^24^ lung cancer,^25^ lung transplant,^26^ post-TB lung disease^27^ and post-COVID-19.^28^ Evidence from high-income countries (HICs) suggests that PR significantly reduces the direct costs of COPD by decreasing healthcare system usage, particularly unplanned hospital admissions.^29^

However, current PR evidence is predominantly based on studies from HICs.^30^ Clinical PR services are not widely available in LMICs,^31^ where significant modifications may be required due to differences in resources, awareness, culture, healthcare configuration and target disease epidemiology.^32^ A systematic review of PR in Southern Africa demonstrated few trials, generally low-quality evidence for efficacy and no published data from Malawi.^3^ Therefore, we conducted a trial of a PR programme among patients with functionally limiting CRDs at Queen Elizabeth Central Hospital (QECH), Blantyre, Malawi. This article reports results of the post-PR qualitative study which aimed to explore patients’ experiences of living with CRDs before and after participating in the PR programme, and their suggestions to improve a future programme.

## Methods

Our published protocol, available elsewhere,^33^ includes detailed information about the study design, participants’ sample size, recruitment process, eligibility criteria and data collection. A summary is presented here.

### Setting

Healthcare facilities in Malawi are organised into primary, secondary and tertiary levels, linked through a referral system. The primary level includes community health centres, the secondary level includes district hospitals and the tertiary level includes four central hospitals, two of which are in the Southern Region, including QECH in Blantyre. QECH is Malawi’s largest tertiary hospital catering for all primary-level and secondary-level facilities for the Southern Region. The hospital includes a Chest Clinic which serves outpatients with chronic cardiac and respiratory conditions, including chronic heart failure and post-TB lung disease, respectively.

### Participants’ recruitment and incentive

A total of 14 protocol-eligible^33^ patients (eight females and six males, aged ≥18 years) presenting with functionally limiting CRD symptoms to the Chest Clinic at QECH were consecutively identified, invited and consented (by TM, BC, MMi, MMa and EChit) to participate in a PR programme at the QECH’s Physiotherapy Department. No participant had participated in PR previously.

Each participant was assured of being provided with transport money to attend each PR session, covering their travel costs both to and from the PR centre. The amount of transport money given to each participant was to be proportionate to each participant’s travel distance and at least equivalent to the amount of transport money each participant would be charged on public transport such as minibus or motorcycle. Each participant was also given an additional 1000 Malawian kwacha as remuneration for their time. Refreshments such as drinking water were provided to participants at the PR sessions.

### Intervention

The PR programme trial was conducted between February and May 2023. To make it locally appropriate, the PR programme’s design incorporated the perspectives of participants of a formative qualitative study,^34^ some of whom also participated in the actual programme.

The PR programme ran for 6 weeks, two times per week, at QECH’s Physiotherapy Department. A team of six physiotherapists (TM, BC, MMi, CF, MMa, LM) and a nurse (EChit) delivered the PR sessions, coordinated by the principal investigator (FMB). Due to limited physiotherapy gymnasium space and busier physiotherapists in the morning, all PR sessions were scheduled in the afternoon (from around 13:00). The sessions were delivered in a group format; participants were divided into two groups of five each, with one group attending the PR sessions on Mondays and Wednesdays and the other group on Tuesdays and Thursdays. Participants were recruited into the PR sessions on a rolling basis. For instance, the programme started with five participants in the first week of which PR sessions were run by all members of the PR delivery team. From the second week onwards, the number of participants increased to 10, and it was at this point that the PR delivery team was divided into two teams of physiotherapists, with one team delivering PR sessions to the ‘Monday and Wednesday’ participant group and the other team delivering PR sessions to the ‘Tuesday and Thursday’ participant group. The PR sessions consisted of an hour of individualised circuit exercise training, with its intensity progression according to individual participant’s improvement over time. The circuit included exercises for both the upper and lower limbs designed to improve aerobic endurance and muscle strength such as dumbbell lifting, stair climbing, treadmill walking, stationary cycling and sit-to-stand. Participants were encouraged to also exercise at their homes. More details on the individualisation and progression of the PR programme can be found in the protocol published elsewhere.^33^

### Data collection

Post-PR semistructured in-depth interviews with individual participants were conducted by TM and MMi (both had prior qualitative research experience) using a topic guide (online supplemental table 1) which was informed by a literature review and received expert input from a Malawian social scientist (FL). It was not pilot tested, but no significant areas for its quality or content improvement were identified as the interviews progressed. The interviews were conducted in the Head of Physiotherapy Department’s office, which was a quiet and private room within the Physiotherapy Department building at QECH, and their durations ranged from 10 to 38 min (22 min on average). All interviews were conducted in Chichewa (most spoken native language) and audio-recorded.

### Data management

The interview audio files were stored on a password-encrypted computer and transcribed verbatim (by FMB) in their original language (Chichewa) to minimise loss of meaning with English translation^35^; only participant’s quotes included in this paper were translated into English at the time of results reporting. The interview data were anonymised at the time of translation and transcription by replacing participants’ names with a number (eg, ‘participant 1’, ‘participant 2’, etc).

### Data analysis

FMB and TM analysed the qualitative data independently but collaboratively using a deductive thematic approach.^36^ Initially, the researchers collaborated to develop a preliminary coding framework. Data coding was performed manually by the researchers separately using an iterative approach across all qualitative data.^36^ The researchers followed the steps outlined by Braun and Clarke,^36^ including independently going through a process of familiarisation with the data by reading and re-reading transcripts while making reflective notes on the literal content, looking closely at words used by participants, interpreting what the data meant by assigning initial codes or classifications to segments of text and exploring relationships between these classifications and developing core themes. The two researchers discussed their separate themes and merged them collaboratively.

### Patient and public involvement

Patients (and their caregivers) were involved in the formative qualitative study for this trial to make sure the design of the PR programme was culturally acceptable for them; data were collected from them through in-depth interviews. At the end of the programme, they were also involved by sharing their experiences, through in-depth interviews, about the programme and suggestions to improve a future PR programme.

## Results

Out of the 14 patients who consented to participate, 4 did not participate: one male patient died of a cardiovascular complication, one female patient relocated to another city and two patients (one male and one female) became unreachable. The remaining 10 patients (six females and four males) participated in the PR programme until completion and were all interviewed post-PR. Their socio-demographic and clinical characteristics are shown in table 1.

**Table 1:** Participants’ socio-demographic and clinical characteristics

Participant’s number	Sex	Age ranges (in years)	Documented respiratory diagnoses in the participants’ hand-held health records	Other documented diagnoses/comorbidities
1	Female	In her 40s	Asthma	Hypertension
2	Female	In her 20s	Post-TB bronchiectasis	HIV
3	Male	In his 40s	Asthma	None
4	Female	In her 60s	Asthma	None
5	Male	In his 50s	COPD, bronchiectasis	HIV
6	Female	In her 40s	Post-TB	HIV
7	Male	In his 80s	Post-TB, COPD	Cor-pulmonale, hypertension
8	Female	In her 40s	Post-TB, asthma	HIV, depression
9	Male	In his 20s	Post-TB lung fibrosis	HIV, depression
10	Female	In her 50s	Asthma, community-acquired pneumonia	HIV

COPD, chronic obstructive pulmonary disease; TB, tuberculosis.

From the analysis, five key themes emerged: (1) debilitating symptom experience of CRD prior to PR; (2) positive impact of PR on living with CRD; (3) contextual programme design improved participants’ experience with PR; (4) one size does not fit all and (5) challenges and opportunities for home-based PR. We now describe these five themes and include their subthemes and select illustrative participants’ quotes in tables 2–5, for example, P1F40s is participant 1, female, in her 40s, etc.

### Theme 1: debilitating symptom experience of CRD prior to PR

Participants described various symptoms of their CRDs, which they said were more severe before they participated in the PR programme. These symptoms included breathlessness, (productive) cough, wheeze, chest tightness, chest pain, dizziness, leg pain, a feeling of body heaviness and body weakness. Before PR, these symptoms affected participants’ physical, psychological and social health negatively, as well as disturbed their sleep. The symptoms were a reason for some participants’ frequent use of inhaler, hospital visits and hospitalisations, although they felt that these were less helpful (table 2).

**Table 2:** Illustrative quotes about living with a chronic respiratory disease before pulmonary rehabilitation

Subtheme	Illustrative quotes
Symptom occurrence	“Upon waking up in the morning, the body used to feel heavy, feeling like sick from malaria and what… to walk, [I used to feel] chest pain, leg pain.” (P2F20s)
Physical health	“Before I started PR, when I walk – I like walking a lot – I used to experience frequent breathlessness. … I used to stop frequently to rest.” (P5M50s)
Psychological health	“The difference [between pre- and post-PR] is that we used to be sedentary [before PR]. We were afraid of doing an activity that we could do, saying ‘If I do this with force, maybe it will worsen my condition.’” (P4F60s)
Social health	“Often, I could only be in one place, struggling with sickness, just sleeping. When my friends were going to parties or to church, I was failing to go because of sickness …” (P1F40s)
Sleep health	“… [before PR], my breathing used to be difficult … difficult especially at night when I sleep. When I sleep, and start coughing, I used to get very breathless, sometimes sweating. So, I could wake up [and] sit down, to do what? To rest, to get back my breath.” (P6F40s)
Medication, hospital visits and hospitalisation	“So, I used to come to the hospital frequently. Getting admitted in 4A [ward], sometimes [getting admitted] up there where there are lots of window glasses [AETC]; I used to be hospitalised there for a long time. Maybe wondering, ‘What is happening? I can’t see anything [the treatment received there] helpful.’ Giving me Jackson [injection], giving me drugs, but nothing was what? Helpful.” (P8F40s)

### Theme 2: positive impact of PR on living with CRD

Participants reported positive impact of the PR programme on various aspects of their lives, including improving their CRD symptoms (table 3). For example, they reported reductions in the magnitude and frequency of the symptoms, accompanied by improvements in their physical, psychological, social and sleep health. In addition, participants reported the acquisition of new and/or improved knowledge, including knowledge about exercise benefits. This knowledge helped them have a positive attitude towards exercises and/or physical activities, including removing their fear of engaging in them. Finally, due to PR, some participants reported using inhalers less frequently, the inhalers became more effective/responsive and that they were visiting the hospital less frequently.

**Table 3:** Illustrative quotes about living with a chronic respiratory disease after pulmonary rehabilitation

Subtheme	Illustrative quotes
Symptom occurrence	“What has changed … on the part of breathlessness, now I am not feeling as breathless as previously [before PR]. I am feeling [nowadays] breathless but occasionally, but I can do my household chores as a woman, being comfortable/relaxed. The breathlessness does not occur to me as frequently as previously.” (P1F40s)
Physical health	“I am proud and happy because my body has now relaxed compared to how I was previously. Because even work, I was unable to do [before PR]; lifting [objects], I couldn’t lift. But now, I can walk up a slight hill, on a flat surface too, walking well, or lifting and doing other household chores like laundry … doing household chores as a woman … while, previously, I couldn’t do so.” (P1F40s)
Psychological health	" But when we I came here [after PR], I put aside many of the anxieties … they [anxieties] ended. … I feel sweet in my life. It’s good, it’s done me good, coming here.” (P8F40s)
Social health	“… these days [after PR], people are proposing me … But at that time [before PR], not even ‘tsiii, tayimani’ [Chichewa words to romantically stop a woman in the streets] … was there. But these days, those with ‘tsii’ are? Increasing. But I think that all that power, or [my new] physical appearance, or the way they see me … because I did not have that power [to catch men’s romantic attention] at that time [before PR], I didn’t have enough strength, I couldn’t walk smoothly or freely like one should do. But since I started this program [PR], I see that things for me have really what? Changed. Because I have the strength …” (P6F40s)
Sleep health	“Uhm, we can say that now it is better because, since the beginning of that program, any strange symptoms … especially breathlessness, it’s no longer happening or at night. Because sometimes … this breathlessness could come when I'm sleeping. Then, I would take the inhaler and puff it. But now, since this started [PR], I have not been breathless at night.” (P5M50s)
Knowledge	“Yes, but this [PR] program is really good because people were afraid that, ‘If I do that, I will be breathless. If I do this, I will breathless’, not knowing that maybe by doing so we are weakening our body a lot. But when we do this [PR], … I have learned that activities are important … a problem like that [breathlessness] can recover better if I do activities. Because many of us live in fear of danger, fearing that we might be breathless in this situation [of doing activities]. So, I have learned that it is important for the [PR] program to continue.” (P10F50s)
Medication, hospital visits and hospitalisation	“…. sometimes, I can do something without taking medicine; I am able to walk or climb, things that can still give us breathlessness, but finding that we are able to climb on that place … the breathlessness will come but it will not be necessary for us to use medicine. It’s as if our bodies are relaxing, slowly relaxing because of those exercises when I take the medicine, I find that the medicine is responding quickly. Yes, that, ‘Ah, let’s take her to the hospital’, no, it [the exacerbation] resolves right there at home [without necessarily going to the hospital].” (P4F60s)

### Theme 3: contextual programme design improved participants’ experience with PR

When asked about what went well and what did not, participants said the programme went well generally. They attributed this to several reasons, including the aforementioned gained PR benefits, good personal attributes of the PR delivery team (such as welcoming, heartwarming/warmful, cheerful, kind, polite, hardworking and participatory or engaging and interactive), transport money provision and good exercise design (such as the group delivery format, exercise individualisation and the right choice of the exercises). For example:

Truth is good. For me, from the first day [of PR] to come again today, everything was good. Yeah, because in this place, you didn't insult us … but you were welcoming us as your children. Pleasant … anything was fun. Problems do not fail everywhere, but for me I saw that everything was a good thing. (P7M80s)

Yes, it [the PR program] also went well because you were providing transport money which made travel [to and from the PR centre] easier because according to the [financial] situation of people like us, maybe we should have started the program and left it on the way because sometimes we don't have money … (P9M20s)

Every aspect has gone well. Yes, because … You were also looking at how sick a patient was, you were treating the patient according to how sick he/she was [exercise individualisation]. (P2F20s)

### Theme 4: one size does not fit all

Participants suggested that, for more people with CRD to benefit from the PR programme, the programme should continue beyond the study, more patients should be recruited and the programme should be widely implemented in Malawi. Some participants suggested that this would be possible with further funding and government support for the programme. In addition, participants suggested the need to raise awareness about the intervention, with themselves even willing to be champions of that awareness. Regarding the timing of the PR sessions, some participants were okay with the afternoon sessions while some participants suggested shifting them from afternoon to morning hours for their convenience. Some participants’ suggestions were about adjusting the exercise design of the PR programme which included increasing the intensity, diversity/variety, number and duration of the exercise stations. They said these adjustments could help them benefit more from the programme. Some participants suggested delivering comprehensive/structured education sessions within the PR programme, including education on safe home-based exercises and self-management skills (table 4).

**Table 4:** Illustrative quotes of suggestions to improve a future pulmonary rehabilitation programme

Subtheme	Illustrative quotes
Programme reach	“… are you also going to reach other districts [with the PR program] or it’s just around here, Blantyre, only? … if yes and people have been well-trained and realise its benefit, I hope that many can benefit. … these [PR program] shouldn’t just start and end, no, but should continue. Because, if you do it on a few people, there are many people who are suffering; maybe even we [current participants] are able to walk, we are coming here … others [with similar conditions] are indoors, they do not know how helpful physical exercises are. So, my opinions are that if the government agrees it [PR program] to be special that a person with such conditions as these should go and do physical exercises, not making us depend on drugs only, I feel like it might help. Of course.” (P2F20s)
Programme awareness	“I can say this: that those who can listen to me, listen … maybe you can throw this [PR] program somewhere with the caption, ‘But so-and-so was helped. She is grateful for this [PR], she was helped. She was lacking good breathing, walking, and activity performance. But when she came to physiotherapy with this disease, it [PR] did her good.’ So that all can listen to a program like this, take a lesson from it, and be encouraged.” (P8F40s)
Timing of the programme sessions	“Uhm maybe time … it needs good timing; you know we are doing it for the sake of others who come from afar. That maybe shifting the afternoon program to be in the morning. … in the afternoon, after you have eaten [lunch], the body gets kind of drunk … when the body is drunk, its actions are lazy-like. While in the morning, what does the body have? It has power. Yes, you can maybe do more exercises than that [than in the afternoon].” (P2F20s)
Exercise design (intensity, diversity/variety, number and duration of the exercise stations)	“And also changing the duration [of a PR session] – I mean, you could have increased it, maybe to 1 and a half hour or even 2 hours, so that people’s [participants’] bodies should remain relaxed [referring to the body relaxation benefit of PR] because most of them [participants], not to lie, maybe … they were doing these physical exercises only here – maybe they don’t do it at home … I think you could have also added more [exercise] stations and other activities [different exercises] so that maybe people’s bodies will relax …” (P9M20s)
Self-management education	“…. You should have also been telling us … helping us … activities, ‘you can do this, do this, do this. When you arrive home, when you see [feel] this [symptoms/exacerbations], also do this.’ Because sometimes when I do it [exercise] at home, sometimes was like I was getting breathless with the exercise when I sit down. So, I don’t know, what can we do in that case? Should we just use the medicine or whatever?” (P10F50s)

### Theme 5: challenges and opportunities for home-based PR

Motivated by PR benefits, participants were willing to continue exercising and/or being physically active at their homes, and some reported already doing so and even teaching others with similar lung conditions. However, most participants mentioned lack of equipment at home as a potential barrier to home-based exercises. On the other hand, potential enablers to home-based exercises and/or physical activities included using locally available equipment for the exercises or doing exercises that do not require any equipment like walking or taking advantage of any available opportunities to stay physically active (table 5).

**Table 5:** Illustrative quotes of perspectives on home-based pulmonary rehabilitation programme

Subtheme	Illustrative quotes
Willingness to continue programme at home	“Yes, I am ready to do everything in readiness to do the physical exercises. Or jogging, or lifting… I can lift like a packet of sugar for the physical exercises, I do. Jogging up a slight hill and down, I also do. Like the way I was doing here, I am doing likewise at home.” (P1F40s)
Motivators for continuing programme at home	“Yes, I would like [to continue the program at home] because it’s helping me … I can do laundry now… yes, it [the program] is helping me. The body is not [no longer] weak [due to the program].” (P2F20s)
Potential barriers to home-based programme	“Yes, I'm happy to continue the program at home but with fewer [exercise] stations, considering the equipment you use [in the hospital-based PR]; because we can't afford a treadmill. I may not be able to afford those weights [dumbbells] … So, continuing [exercising at home] can be there … but the resources to do so are the ones that may be missing. But I wish to continue, I can do it, but not according to the way you [PR delivery team] were doing it [at the hospital].” (P9M20s)
Potential enablers to home-based programme	“But the other ones [some PR equipment], we can make, like those dumbbells … pouring sand, they told us, in bottles, it’s possible. Sit to standing … that’s also possible – some things were possible.” (P10F50s)

## Discussion

Participants in this study reported negative experiences of living with CRD before participating in a PR programme. Those negative experiences included poor physical health (including limitations in performing activities of daily living), poor psychological health (including anxiety), poor social health (including social exclusion and discrimination), poor sleep health (including sleep disturbance) and frequent inhaler use, hospital visits and admissions. Similar experiences were reported by people with CRDs in Sudan and Tanzania.^37^ After participating in the PR programme, participants reported improved experiences of CRD in all the areas above, like those in Uganda.^38^

CRDs pose an economic burden on patient and their households in Malawi. For example, chronic cough was found to be associated with unaffordable health-seeking costs among 608 people with CRDs (including post-TB lung disease, asthma, COPD and bronchiectasis) in a cross-sectional community-based survey conducted in rural Malawi.^39^ These costs were mainly influenced by costs of transport and drugs. Therefore, the improvements in our participants’ physical activity performance and return to work, plus their reduced frequency of inhaler or medication use and hospital visits, may suggest a potentially positive economic impact of PR in Malawi for both patients and healthcare system. However, the economic evaluation of PR in Malawi is yet to be conducted. In HICs, PR has proven to reduce patient and health system costs owing to reductions in exacerbations, medical visits, hospitalisation rates and length of stay.^40 41^

The participants’ positive experiences of the PR programme and some participants’ suggestion to include self-management education in the programme to improve it suggest that PR improvements in CRD symptoms (eg, breathlessness, cough and body weakness), physical issues (eg, a sense of physical well-being such as body relaxation and strength, performance of activities of daily living and return to work), social issues (eg, ability to interact with others and participate in social or group events), psychological issues (a sense of peace of mind and reduction in anxiety), sleep quality, health literacy, medicine use, hospital visits and hospitalisation might be important patient-reported outcomes for PR in the Malawian population but need further investigation such as through a core outcome set study.

It is noteworthy that the PR delivery team in this study focused on delivering exercise training to participants during the PR sessions. Some participants’ suggestion in the current study that self-management education would have been included in the completed PR programme echoes that suggests that health literacy or knowledge about their disease (including self-management) might be an important PR outcome in patients with CRDs in this setting. Self-management education is particularly recommended in WHO’s package of essential rehabilitation interventions for patients with COPD.^16^ Our primary focus on delivering the exercise training component was based on evidence (largely from HICs) which presents exercise training as a cornerstone of PR that must be prioritised^16^ and, more recently, as one of the ‘essential’ components of PR as it is underpinned by strong evidence.^42^ On the other hand, structured education, self-management training and smoking cessation support were found to be ‘desirable’ (not essential) components of PR because strong evidence of their individual impacts is not yet available.^42^

Barriers to PR commonly reported in LMICs and HICs include travel and transport difficulties.^43 44^ Therefore, transport money provision was unsurprisingly mentioned by participants of the current study as one of the PR programme’s success factors, facilitating their attendance and completion of the programme. However, providing transport money to patients in Malawi is currently not a sustainable strategy to ensure long-term PR uptake at a hospital due to high economic challenges in the country (the current study was funded to provide participants with transport money, which is not the case in the routine clinical practice). Therefore, our finding reinforces the growing call for alternative models of PR delivery to be investigated, including home-based and community-based PR, to address the travel and transport barrier, especially in Africa.^43^

Encouragingly, participants of the current study expressed enthusiasm for home-based PR, and some were already continuing to exercise at home. Participants suggested that a safe and effective home-based PR programme would be possible with a therapist-prescribed home exercise programme, education on self-management skills and use of locally available exercise equipment. In a randomised, controlled equivalence trial in Australia, a home-based PR programme, using minimal resources (including readily available equipment in the patients’ home environment such as sit to stand from a dining chair and water bottles or bags of rice for upper limb weights) and little direct supervision, delivered short-term improvements in clinical outcomes (functional exercise capacity and health-related quality of life) that were equal to or greater than those seen in a conventional centre-based PR programme in people with COPD.^45^ However, the effectiveness and participants’ experiences of a home-based PR model in LMICs, including Malawi, need to be empirically investigated as they may differ from HICs’.

Participants also mentioned good personal attributes of the PR delivery team (such as welcoming, heartwarming/warmful, cheerful, kind, polite, hardworking and engaging or interactive) as one of the PR programme’s success factors. These therapist attributes are likely to contribute to a strong therapeutic alliance, which is essential for increased patient compliance to treatment and subsequent improved patient outcomes.^46 47^ However, the therapeutic alliance concept is yet to be extensively explored in the field of PR; few available PR studies have focused on patients’ (not therapists’) personality traits or characteristics.^48 49^

The inconvenient timing of PR sessions is a known barrier to attending PR.^44^ However, some participants’ suggestion to shift PR sessions from afternoon to morning hours for their convenience in this study highlights the current challenge of providing PR that meets individual patients’ needs in Malawi. The demand for rehabilitation services in the country is high. Yet, the rehabilitation centres (including QECH, study setting) are currently understaffed, underequipped and less spacious to serve many patients efficiently. We chose afternoon hours for the PR sessions due to some of these reasons. Fortunately, no participant dropped out of the study or skipped a PR session due to this timing. However, we joined participants’ call for further funding and government support towards rehabilitation services (including PR) if patients’ rehabilitation needs are to be met. This aligns with the WHO ‘Rehabilitation 2030: a Call for Action’ initiative.^15^

## Study strengths and limitations

This is the first acceptability study of PR in Malawi and responds to other researchers’ calls for PR in Malawi.^5 50^ But the theoretical transferability^51^ of our findings is limited by heterogeneous study participants (eg, various CRDs and comorbidities among participants including HIV infection), as well as the study having been conducted at a single site with a relatively small sample of patients. In addition, in the context of no provision of transport money, attendance and completion rates might differ. Translated participants’ quotes from Chichewa to English might have lost the original meaning.^15^ Interviews were conducted by TM and MMi, who were also involved in delivering PR sessions; this might have influenced participants’ responses during interviews. However, interview transcription and data analysis were led by the principal investigator (FMB), who had not been involved in both delivery of PR sessions and interviews.

## Conclusion

The PR programme alleviated participants’ negative impacts of CRD on their physical, social, psychological and sleep health, associated with frequent inhaler use, hospital visits and admissions. In thinking about scaling PR within the Malawian context, key challenges need to be considered, including PR financing, access to care in terms of transport and timing, task-shifting to primary care and community, and balancing equipment to optimise intervention quality with sustainability beyond the current PR programme.

## Supplementary material

10.1136/bmjresp-2024-002547 online supplemental table 1

## Data Availability

Data are available upon reasonable request.

## References

[1] Meghji J, Mortimer K, Agusti A (2021). Improving lung health in low-income and middle-income countries: from challenges to solutions. Lancet.

[2] van Kampen SC, Wanner A, Edwards M (2018). International research and guidelines on post-tuberculosis chronic lung disorders: a systematic scoping review. BMJ Glob Health.

[3] Bickton FM, Fombe C, Chisati E (2020). Evidence for pulmonary rehabilitation in chronic respiratory diseases in sub-Saharan Africa: a systematic review. Int J Tuberc Lung Dis.

[4] Nightingale R, Jary H, Meghji J (2020). Non-communicable respiratory disease in Malawi: a systematic review and meta-analysis. Malawi Med J.

[5] Meghji J, Lesosky M, Joekes E (2020). Patient outcomes associated with post-tuberculosis lung damage in Malawi: a prospective cohort study. Thorax.

[6] Meghji J, Gregorius S, Madan J (2021). The long term effect of pulmonary tuberculosis on income and employment in a low income, urban setting. Thorax.

[7] Soriano JB, Kendrick PJ, Paulson KR (2020). Prevalence and attributable health burden of chronic respiratory diseases, 1990–2017: a systematic analysis for the Global Burden of Disease Study 2017. Lancet Respir Med.

[8] GOLD (2018). Global strategy for diagnosis, management and prevention of chronic obstructive pulmonary disease (2018 report).

[9] GINA (2018). 2018 GINA report, global strategy for asthma management and prevention.

[10] Stolbrink M, Thomson H, Hadfield RM (2022). The availability, cost, and affordability of essential medicines for asthma and COPD in low-income and middle-income countries: a systematic review. Lancet Glob Health.

[11] Kibirige D, Sanya RE, Nantanda R (2019). Availability and affordability of medicines and diagnostic tests recommended for management of asthma and chronic obstructive pulmonary disease in sub-Saharan Africa: a systematic review. Allergy Asthma Clin Immunol.

[12] Eisner MD, Iribarren C, Blanc PD (2011). Development of disability in chronic obstructive pulmonary disease: beyond lung function. Thorax.

[13] Morgan MDL, Britton JR (2003). Chronic obstructive pulmonary disease 8: non-pharmacological management of COPD. Thorax.

[14] Cieza A, Causey K, Kamenov K (2021). Global estimates of the need for rehabilitation based on the Global Burden of Disease study 2019: a systematic analysis for the Global Burden of Disease Study 2019. Lancet.

[15] Gimigliano F, Negrini S (2017). The World Health Organization “Rehabilitation 2030: a call for action”. Eur J Phys Rehabil Med.

[16] World Health O (2023). Package of interventions for rehabilitation: module 4: cardiopulmonary conditions: web annex: literature reviews and evidence tables.

[17] Rochester CL, Alison JA, Carlin B (2023). Pulmonary Rehabilitation for Adults with Chronic Respiratory Disease: An Official American Thoracic Society Clinical Practice Guideline. Am J Respir Crit Care Med.

[18] Spruit MA, Singh SJ, Garvey C (2013). An official American Thoracic Society/European Respiratory Society statement: key concepts and advances in pulmonary rehabilitation. Am J Respir Crit Care Med.

[19] Bolton CE, Bevan-Smith EF, Blakey JD (2013). British Thoracic Society guideline on pulmonary rehabilitation in adults. Thorax.

[20] Dowman L, Hill CJ, May A (2021). Pulmonary rehabilitation for interstitial lung disease. Cochrane Database Syst Rev.

[21] Morris NR, Kermeen FD, Jones AW (2023). Exercise-based rehabilitation programmes for pulmonary hypertension. Cochrane Database Syst Rev.

[22] Osadnik CR, Gleeson C, McDonald VM (2022). Pulmonary rehabilitation versus usual care for adults with asthma. Cochrane Database Syst Rev.

[23] Radtke T, Nevitt SJ, Hebestreit H (2017). Physical exercise training for cystic fibrosis. Cochrane Database Syst Rev.

[24] Lee AL, Gordon CS, Osadnik CR (2021). Exercise training for bronchiectasis. Cochrane Database Syst Rev.

[25] Granger CL, McDonald CF, Berney S (2011). Exercise intervention to improve exercise capacity and health related quality of life for patients with Non-small cell lung cancer: a systematic review. Lung Cancer.

[26] Langer D (2015). Rehabilitation in Patients before and after Lung Transplantation. Respiration.

[27] Ando M, Mori A, Esaki H (2003). The effect of pulmonary rehabilitation in patients with post-tuberculosis lung disorder. Chest.

[28] Vieira A da S, Pinto A, Garcia B (2022). Telerehabilitation improves physical function and reduces dyspnoea in people with COVID-19 and post-COVID-19 conditions: a systematic review. J Physiother.

[29] Johnston K, Grimmer-Somers K (2010). Pulmonary rehabilitation: overwhelming evidence but lost in translation?. Physiother Can.

[30] Habib GMM, Rabinovich R, Divgi K (2020). Systematic review of clinical effectiveness, components, and delivery of pulmonary rehabilitation in low-resource settings. NPJ Prim Care Respir Med.

[31] Singh SJ, Halpin DMG, Salvi S (2019). Exercise and pulmonary rehabilitation for people with chronic lung disease in LMICs: challenges and opportunities. Lancet Respir Med.

[32] Habib GM, Uzzaman MN, Malik P (2020). Engaging with stakeholders in a research programme to promote implementation of pulmonary rehabilitation in Bangladesh: Challenges and opportunities. J Glob Health.

[33] Bickton FM, Mankhokwe T, Nightingale R (2022). Protocol for a single-centre mixed-method pre-post single-arm feasibility trial of a culturally appropriate 6-week pulmonary rehabilitation programme among adults with functionally limiting chronic respiratory diseases in Malawi. BMJ Open.

[34] Bickton FM, Mankhokwe T, Mitengo M (2022). “My life is not going to be the same, my health is going to improve”: a cross-sectional qualitative study of patients’ experiences of living with chronic respiratory symptoms and their views on a proposed pulmonary rehabilitation program at Queen Elizabeth Central Hospital, Blantyre, Malawi. Wellcome Open Res.

[35] van Nes F, Abma T, Jonsson H (2010). Language differences in qualitative research: is meaning lost in translation?. Eur J Ageing.

[36] Braun V, Clarke V (2006). Using thematic analysis in psychology. Qual Res Psychol.

[37] Egere U, Shayo EH, Chinouya M (2023). “Honestly, this problem has affected me a lot”: a qualitative exploration of the lived experiences of people with chronic respiratory disease in Sudan and Tanzania. BMC Public Health.

[38] Jones R, Muyinda H, Nyakoojo G (2018). Does pulmonary rehabilitation alter patients’ experiences of living with chronic respiratory disease? A qualitative study. Int J Chron Obstruct Pulmon Dis.

[39] Sichali JM, Khan JAK, Gama EM (2020). Direct costs of illness of patients with chronic cough in rural Malawi—Experiences from Dowa and Ntchisi districts. PLoS ONE.

[40] Atsou K, Crequit P, Chouaid C (2016). Simulation-Based Estimates of the Effectiveness and Cost-Effectiveness of Pulmonary Rehabilitation in Patients with Chronic Obstructive Pulmonary Disease in France. PLoS One.

[41] Mosher CL, Nanna MG, Jawitz OK (2022). Cost-effectiveness of Pulmonary Rehabilitation Among US Adults With Chronic Obstructive Pulmonary Disease. *JAMA Netw Open*.

[42] Holland AE, Cox NS, Houchen-Wolloff L (2021). Defining Modern Pulmonary Rehabilitation. An Official American Thoracic Society Workshop Report. Ann Am Thorac Soc.

[43] Bickton FM, Shannon H (2022). Barriers and Enablers to Pulmonary Rehabilitation in Low- and Middle-Income Countries: A Qualitative Study of Healthcare Professionals. Int J Chron Obstruct Pulmon Dis.

[44] Keating A, Lee A, Holland AE (2011). What prevents people with chronic obstructive pulmonary disease from attending pulmonary rehabilitation? A systematic review. Chron Respir Dis.

[45] Holland AE, Mahal A, Hill CJ (2017). Home-based rehabilitation for COPD using minimal resources: a randomised, controlled equivalence trial. Thorax.

[46] Leach MJ (2005). Rapport: a key to treatment success. Complement Ther Clin Pract.

[47] Ackerman SJ, Hilsenroth MJ (2003). A review of therapist characteristics and techniques positively impacting the therapeutic alliance. Clin Psychol Rev.

[48] Caille P, Alexandre F, Molinier V (2021). The role of personality traits in inpatient pulmonary rehabilitation response in patients with chronic obstructive pulmonary disease. Respir Med.

[49] Hayton C, Clark A, Olive S (2013). Barriers to pulmonary rehabilitation: characteristics that predict patient attendance and adherence. Respir Med.

[50] Nightingale R, Chinoko B, Lesosky M (2022). Respiratory symptoms and lung function in patients treated for pulmonary tuberculosis in Malawi: a prospective cohort study. Thorax.

[51] Korstjens I, Moser A (2018). Series: Practical guidance to qualitative research. Part 4: Trustworthiness and publishing. Eur J Gen Pract.

