# Supplementary material for: Acceptability of pulmonary rehabilitation in Malawi: a qualitative study
Source: BMJ Open Respir Res. 2025 May 22;12(1):e002547. doi: 10.1136/bmjresp-2024-002547 (PMC12096972; doi:10.1136/bmjresp-2024-002547)
Supplement: online supplemental table 1 [file bmjresp-12-1-s001.docx]

**Supplementary Table: Semi-structured topic guide for post-PR in-depth interviews**

| **Introduction:**  *Thank you for participating and completing a PR program in the study that has just ended. Now, this part of the study aims to establish your experiences with the program including its impact on your health or quality of life, your views on what went well and what went wrong with the program, your recommendations on how to improve the program, and your motivation/willingness to undertake the program again in the future and to continue it at your home. Please, note that there aren’t any right or wrong answers. I am just trying to gather your opinions and experiences. I am asking the same questions to your friends with whom you undertook the program and the information you share with me is confidential.*   1. **Impact of the PR program on participants:**    1. How are you feeling now?    2. How does your disease affect you since you taking part in the rehabilitation program?    3. What has changed since the last time I spoke to you?    4. Which aspects of your activities have changed?    5. How do you now feel about taking part in activities such as going out/walking, etc.?    6. How do you feel about your breathlessness now? 2. **Recommendations**     1. What do you think went well and what did not go well with the program?    2. What recommendations can you suggest improving the program?    3. In the future, for you to attend and complete a PR program, what circumstances would have to change for you? 3. **Maintenance of PR program**     1. Would you consider continuing the program at your home? 4. **Conclusion**     1. Are there any other issues you would like to raise? |
| --- |
